# Hippocrates Redivivus

**Published:** 1878-07

**Authors:** 


Hippocrates Redivivus.—Dr. W. P. King, at the late meeting of the Missouri State Society, got off the best thing we have seen for many a day. He was relating his early experience, and said:

"Of all the things that afflicted me in my early experience as a physician, the idea of a consultation was the worst. I dreaded meeting the old physicians. I thought gray hairs and wisdom were synonymous words. There was a man practicing in that country who had been there, well, ever since the flood. He had a wonderful reputation. I put the thing off as long as I could, but the inevitable came at last. One of the prominent men in my neighborhood got sick with pneumonia, and when the lung had solidified, and he had passed near unto death, I honestly informed the family of his dangerous condition. Of course they wanted Smith. I call him Smith, because that was not his name. Smith came, and I met him with fear and trembling. He wore a No. 3 hat and a No. 13 boot. I gave him a history of my case and the treatment, and awaited the coming storm. I expected to be overwhelmed with a flood of technicalities and with his wonderful knowledge of pathological anatomy. He looked at me through his spectacles with the gravity of an owl, and said, 'Doctor, did you ever try a black catskin poultice in these cases?' I admitted that, in my ignorance, I never had. I thought it a good time for me to get out of the case, so I informed the family that, as Dr. Smith had been their family physician before they knew me, he had better take the case. They were only too glad to make the change. That was an awful and calamitous night for cats, especially black cats.

"The man died, and Smith told them that if he had seen the case two hours earlier, and had succeeded in getting a 'leetle' blacker cat, he would have saved him. The family still think so." —*Louisville Med. News.*

